# Association between glial fibrillary acidic protein, glial-derived neurotrophic factor, and fatty acid-binding protein-2 at birth in the incidence of necrotizing enterocolitis in preterm infants

**DOI:** 10.3389/fped.2022.1010013

**Published:** 2022-10-20

**Authors:** Dina Angelika, Risa Etika, Munawaroh Fitriah, Naomi Nathania Kusumawardani, Angelica Diana Vita, Roedi Irawan, Kian Djien Liem, I Dewa Gede Ugrasena

**Affiliations:** ^1^ Doctoral Program of Medical Science, Faculty of Medicine Universitas Airlangga, Surabaya, Indonesia; ^2^ Department of Child Health, Faculty of Medicine Universitas Airlangga, Surabaya, Indonesia; ^3^ Department of Clinical Pathology, Faculty of Medicine Universitas Airlangga, Surabaya, Indonesia; ^4^ Medical Program, Faculty of Medicine Universitas Airlangga, Surabaya, Indonesia; ^5^ Department of Neonatology, Radboud University Medical Center, Nijmegen, The Netherlands

**Keywords:** glial fibrillary acidic protein, glial-derived neurotrophic factor, fatty acid-binding protein-2, preterm, necrotizing enterocolitis

## Abstract

**Background:**

This study aimed to analyze the relationship between glial fibrillary acidic protein (GFAP), glial-derived neurotrophic factor (GDNF), and fatty acid-binding protein-2 (FABP-2) in preterm infants on the incidence of NEC.

**Methods:**

Preterm infants with a birth weight <1,500 g and gestational age <34 weeks were included in this study. Biomarker examination was performed using the umbilical vein blood at birth (first sample). Biomarker examination was repeated if the infant developed symptoms of NEC using peripheral vein blood (second sample). Infants were observed for 14 days. If NEC did not exist, a biomarker examination was performed at 14 days.

**Results:**

This study included 30 preterm infants, nine infants experienced NEC. The values of GFAP, GDNF, and FABP-2 (median and range) in the group with NEC were higher than those in the group without NEC in both the first samples {GFAP [1.40 (0.20–6.50) vs. 0.30 (0.10–1.30) *P* = 0.014], GDNF [2.84 (1.05–14.11) vs. 1.56 (1.07–3.48) *P* = 0.050], and FABP-2 [621.70 (278.40–2,207.00) vs. 294.20 (211.40–597.50) *P* = 0.002]} and second samples {GFAP [2.40 (0.30–3.10) vs. 0.30 (0.10–0.60) *P* = 0.003], GDNF [2.99 (0.56–10.30) vs. 1.46 (0.85–2.24) *P* = 0.019], and FABP-2 [646.8 (179.20–1,571.00) vs. 314.90 (184.70–521.60) *P* = 0.040]}. In infants with NEC, the median values of GFAP [2.40 (0.30–3.10) vs. 1.40 (0.20–6.50) *P* = 0.767], GDNF [2.99 (0.56–10.30) vs. 2.84 (1.05–14.11) *P* = 0.859], and FABP-2 [646.80 (179.20–1,571.00) vs. 621.70 (278.40–2,207.00) *P* = 0.953] in the second sample were higher than those in the first sample. Logistic regression demonstrated that GFAP at birth (Odds Ratio [OR] = 15.629, 95% Confidence Interval [CI] = 1.697–143.906, *P* = 0.015) and FABP-2 levels at birth (OR = 1.008, 95% CI = 1.001–1.015, *P* = 0.033) were significantly associated with an increased risk of NEC.

**Conclusion:**

Increased GFAP, GDNF, and FABP-2 at birth are associated with NEC occurrence within two weeks of birth. These findings suggest that early-onset NEC is associated with intestinal injury that occurs during the perinatal or even prenatal period.

## Introduction

Premature birth is the leading cause of death worldwide in children under five. It is estimated that each year, 15% of all deliveries are premature, and most preterm infant deaths are caused by complications of prematurity (1, 2). Necrotizing enterocolitis (NEC) is an inflammatory-necrosis injury of the digestive tract that often occurs in preterm infants with high morbidity and mortality (3). Surgery is required in 40% of NEC cases, although the availability of pediatric surgeons may be limited in low-resource countries (4). There is an association between NEC and adverse neurodevelopmental effects that impair quality of life (5). Despite the discovery of clinical manifestations of NEC in 1965, the mortality and morbidity of NEC are still high. The pathogenesis is still not completely clear and may contribute to its burden (6).

The gastrointestinal tract has a nervous system known as the enteric nervous system (ENS) which is located in the myenteric plexus. An increase in glial fibrillary acidic protein (GFAP) expression was observed in the intestinal myenteric plexus with NEC (7, 8). On the other hand, GFAP reflects the death of astrocytes released into the circulation after blood-brain barrier breakdown, which occurs in hypoxic-ischemic events. Elevated GFAP levels in cord serum correlate with the severity of hypoxic-ischemic encephalopathy (9). There is evidence of an association between brain damage and NEC. A previous study demonstrated that early intervention in NEC can reduce the severity of brain damage (10).

The expression of GFAP and glial-derived neurotrophic factor (GDNF) in colon biopsies was increased in patients with colitis (11). There is evidence that ischemia-reperfusion (I/R) conditions can stimulate the activation of the glial-derived neurotrophic factor GDNF. GDNF activation plays a role in improving intestinal barrier function (12). Studies in mice have shown that GDNF activation can inhibit gut glial cell apoptosis and increase intestinal permeability (13). The key role of GDNF is to maintain the integrity of the intestinal epithelium and to inhibit inflammatory processes (14).

Fatty acid-binding protein-2 (FABP-2) is located on the cell membrane of enterocytes, plays a role in fat transport, has a small molecular mass, and therefore allows translocation into the blood circulation in the event of intestinal damage. Besides being used to detect the presence of NEC, FABP-2 can accurately predict the extent of intestinal damage in NEC (15). To the best of our knowledge, the relationship between GFAP, GDNF, and FABP-2 in NEC patients, which preterm infants often experience, is unclear. Previous studies have reported the presence of hypoxic-ischemic NEC in which the pathogenesis process begins prenatally (16). The presence of early-onset NEC associated with intestinal injury occurring during the perinatal period has not been studied further. Therefore, this study aimed to analyze the relationship between GFAP, GFAP, and FABP-2 levels in preterm infants on the incidence of early-onset NEC.

## Material and methods

### Study design and ethical approval

This observational study was conducted in the neonatal intensive care unit of Dr. Soetomo Hospital, Surabaya, Indonesia, from January 2021 to January 2022. The Ethics Committee of Dr. Soetomo Hospital approved this study with reference number 0123/KEPK/I/2021. Written informed consent was obtained from each parent or representative guardian prior to the study.

### Participant

**Figure 1:**
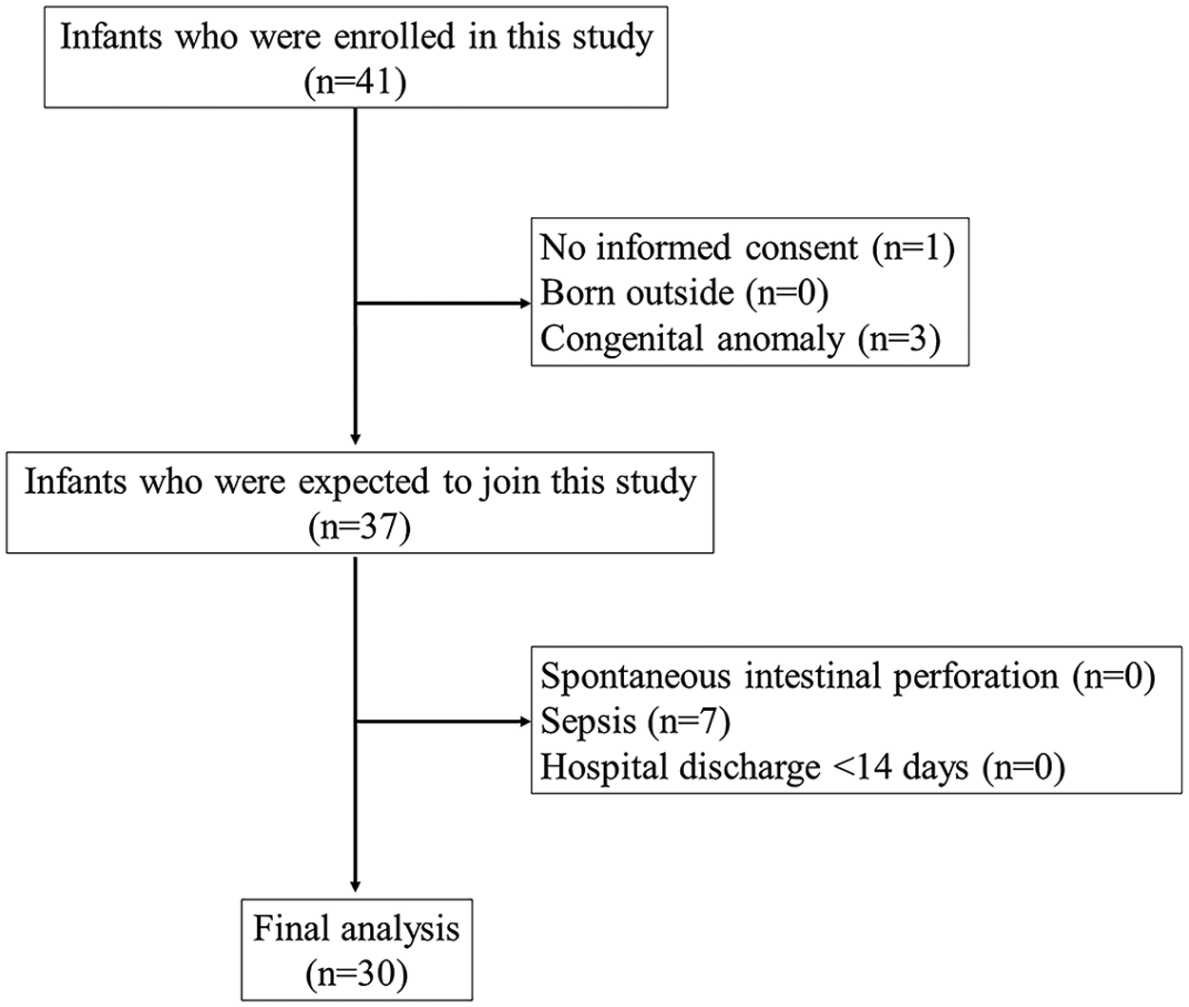
The study flow chart of participant recruitment.

All hospitalized preterm infants with a birth weight <1,500 g and gestational age <34 weeks from January 2021 to January 2022 were included in this study. If the parent or guardian did not sign the consent form, multiple congenital abnormalities were found, or the baby was born outside the hospital, then the baby was excluded. Other exclusion criteria were discharged from the hospital for less than 14 days, sepsis, and diagnosed with spontaneous intestinal perforation (SIP). Infants were monitored for 14 days after birth. In addition, infants who depicted symptoms of NEC were examined using abdominal x-rays. The study flow chart of participant recruitment is shown in Figure 1.

### Necrotizing enterocolitis

In this study, the diagnosis of NEC was established using the modified Bell criteria. Infants included in this study were infants who met the modified Bell criteria stage 2 or higher. The gastrointestinal symptoms observed included abdominal distension, vomiting, increased gastric aspiration >20%, bloody stools, and abdominal tenderness. Abnormal abdominal radiographs revealed dilated bowel, ileus, pneumatosis intestinalis, persistent bowel loops, portal vein gas, and the presence of free air (6). SIP was diagnosed when a primary focal gastrointestinal perforation without signs of multiple inflammation in other intestinal regions was found during surgery (17). Biomarker and radiological examinations were performed by competent, blinded specialists who were not involved in this study. The diagnosis of NEC was established by a senior neonatologist who was not involved in this study.

### Nutrition protocol

Each infant was placed on an umbilical vein catheter (4 Fr polyvinyl chloride umbilical catheter, Vygon; Ecouven, France) for parenteral nutrition. Parenteral nutrition contains dextrose, amino acids, and lipids. In addition, fat-soluble vitamins, water-soluble vitamins, and electrolytes (potassium, sodium, calcium, phosphate, and magnesium) were also added to parenteral nutrition. Amino acids were given in a dose of 2–3.5 g/kg/day, lipids were given in a dose of 1–3 g/kg/day, and dextrose was given in a dose of 6–15 g/kg/day. Enteral nutrition was given through trophic feeding at a dose of 10 ml/kg/day using human milk or preterm formula if human milk was not available. If enteral nutrition was well tolerated, the volume was increased by 20 ml/kg/day to 180 ml/kg/day (18).

### Data collection

Immediately after the infant was born, the placenta was handed over to the pediatrician in a sterile manner and placed in a sterile container. Furthermore, the umbilical vein blood collection was carried out on the placenta as much as 1 ml. The blood was collected in a vacutainer serum separator tube (Becton Dickinson; Franklin Lakes, United States). The biomarker examination was repeated if the infant developed symptoms of NEC within 24 h after the onset of NEC, which was performed using a 1 ml blood obtained from a peripheral vein. The sample was centrifuged at 3,000 rpm for 15 min to obtain the supernatant. Furthermore, the supernatant was stored in a refrigerator at −80 °C until the time of the assay. The clinical pathology laboratory team examined biomarkers (GFAP, GDNF, FABP-2) using the enzyme-linked immunosorbent assay method (Elabscience Biotechnology; Texas, United States). Biomarker examination was carried out according to the manufacturer's procedures. The biomarker sample examined at birth was referred to as the first sample. In contrast, the biomarker sample re-examined when showing symptoms of NEC was referred to as the second sample. In infants who did not describe the symptoms of NEC, the second sample was performed at 14 days.

The variables collected included sex, birth weight, gestational age, NEC, Apgar score <7 at 5 min of age (19), mode of delivery, mortality, maternal history, GFAP level, GDNF level, and FABP-2 level. Maternal history data included preeclampsia, eclampsia, premature rupture of membranes, placenta accrete, respiratory distress, and congenital heart disease. Small for gestational age (SGA) was defined when birth weight was below the 10th percentile (20). This study categorized the mode of delivery into a spontaneous and cesarean section. Furthermore, the variables were classified as groups with NEC and without NEC.

### Statistical analysis

Quantitative variables were described using mean, median, minimum, maximum, and standard deviation (SD), while qualitative variables were described using numbers and percentages. Intergroup comparisons were analyzed using *χ*^2^ test or *t*-test. Normality test was performed using Shapiro-Wilk, the data was considered as not normally distributed when *P*-value < 0.05. If the data was not normally distributed, the median differences were analyzed using Wilcoxon Signed Rank test or Mann–Whitney *U* test. Correlation analysis was performed using Spearman correlation. The results would be obtained using correlation coefficient numbers based on the correlation test. This study used logistic-regression tests to analyze each biomarker on the risk of NEC. All statistical analyzes were performed using IBM SPSS Statistics 21 (IBM Corp., Armonk, NY, United States). *P*-value < 0.05 was considered statistically significant.

## Results

### Characteristics of infants with NEC and without NEC

Of 30 infants included in this study, 9 (30%) infants experienced NEC. Most of the infants with NEC were male as many as 5 (55.6%) infants, gestational age 30–<34 weeks as many as 6 (66.7%) infants, birth weight 1,000–<1,500 g as many as 5 (55.6%) infants, and had a history of cesarean delivery as many as 8 (88.9%) infants. There were 4 infants who were SGA, of which 3 with NEC and 1 without NEC. There was no significant difference in SGA infant in the presence of NEC. All NEC infants in this study were singleton pregnancies and had a history of Apgar score <7 at 5 min. The results of statistical analysis showed that maternal history was not significantly associated with NEC in preterm infants in this study. This study also found that birth weight <1,000 g (Odds Ratio [OR] = 16.000, 95% Confidence Interval [CI] = 1.451–176.451, *P* = 0.019) and Apgar score < 7 at 5 min (OR = 0.400, 95% CI = 0.215–0.743, *P* = <0.001) were significantly associated with NEC.

The birth weight of infants with NEC had a median of 1,000 (range = 950–1,450) g and a mean of 1,116.7 (SD = 183.7) g; meanwhile the gestational age of infants with NEC had a median of 31 (range = 27–33) weeks and a mean of 30.6 (SD = 2.4) weeks. The birth weight of infants without NEC had a median of 1,400 (range = 900–1,450) g and a mean of 1,300 (SD = 152.5) g; meanwhile the gestational age of infants without NEC had a median of 30 (range = 28–33) weeks and a mean of 30.7 (SD = 1.7) weeks. The group of infants with NEC had a statistically significant lower birth weight than the group of infants without NEC (*P* = 0.025), but in contrast, there was no significant difference in gestational age (*P* = 0.965). The detailed characteristics of infants with NEC and without NEC are described in Table 1.

**Table 1:** Characteristics of infants with NEC and without NEC.

Variable	With NEC (*n* = 9)	Without NEC (*n* = 21)	*P*-value
	*n* (%)	*n* (%)	
**Sex**			
Male	5 (55.6)	10 (47.6)	0.690^a^
Female	4(44.4)	11 (52.4)	
**Gestational age (weeks)**			
Median (range)	31 (27–33)	30 (28–33)	0.965^b^
Mean (SD)	30.6 (2.4)	30.7 (1.7)	
30–<34	6 (66.7)	17 (80.9)	0.397^a^
27–<30	3 (33.3)	4 (19.1)	
**Birth weight (g)**			
Median (range)	1,000 (950–1,450)	1,400 (900–1,450)	0.025^b^^,^*
Mean (SD)	1,116.7 (183.7)	1,300 (152.5)	
1,000–<1,500	5 (55.6)	20 (95.2)	0.008^a^^,^*
750–<1,000	4 (44.4)	1 (4.8)	
**Small for gestational age**			
Yes	3 (33.3)	1 (4.8)	0.069
No	6 (66.7)	20 (95.2)	
**Mode of delivery**			
Spontaneous	1 (11.1)	5 (23.8)	0.426
Cesarean section	8 (88.9)	16 (76.2)	
**Number of parity**			
Single	9 (100)	18 (85.7)	0.232
Multiple	0 (0)	3 (14.3)	
**Apgar score <7 at 5 min**			
Yes	9 (100)	6 (28.6)	<0.001*
No	0 (0)	15 (71.4)	
**Outcome**			
Death	2 (22.2)	4 (19.1)	0.842
Survive	7 (77.8)	17 (80.9)	
**Maternal history**			
Preeclampsia	3 (20)	12 (80)	0.468
Eclampsia	1 (50)	1 (50)	
Premature rupture of membrane	0 (0)	1 (100)	
Placenta accreta	1 (100)	0 (0)	
Respiratory distress	4 (44.4)	5 (55.6)	
Congenital heart disease	0 (0)	1 (100)	
No data	0 (0)	1 (100)	

Data was presented in number and percentage; SD, standard deviation; NEC, necrotizing enterocolitis.

^a^ Statistical analysis using *χ*^2^ test.

^b^ Statistical analysis using Mann-Whitney *U* test.

* Significant if *P*-value < 0.05.

### Characteristics of infants with NEC

A total of 2 (22.2%) infants out of 9 infants with NEC died. No infant had Bell stage 3B, so there were no cases of surgical NEC. In the group of infants with NEC, there were 3 (33.3%) infants with SGA and 7 (77.8%) infants with a history of cesarean section delivery. Most of the maternal history was respiratory distress as many as 4 (44.4%) mothers. The onset of NEC was seen on a median of 10 (range = 7–12) days after birth and a mean of 10.3 (SD = 1.5) days after birth. The detailed characteristics of infants with NEC are reported in Table 2.

**Table 2:** Characteristics of infants with NEC.

Sample	BW (g)	GA (weeks)	SGA	Onset (day after birth)	Grade	MOD	Maternal history	Outcome
Sample#1	1,200	33	Yes	11	2A	Cesarean section	Preeclampsia	Survive
Sample#2	1,000	32	Yes	7	3A	Cesarean section	Preeclampsia	Died
Sample#3	950	27	No	10	2A	Cesarean section	Placenta accreta	Survive
Sample#4	1,450	31	No	10	2B	Cesarean section	Respiratory distress	Survive
Sample#5	950	33	Yes	10	3A	Cesarean section	Respiratory distress	Died
Sample#6	1,350	33	No	12	3A	Cesarean section	Respiratory distress	Survive
Sample#7	1,150	30	No	11	3A	Cesarean section	Respiratory distress	Survive
Sample#8	1,000	28	No	12	3A	Spontaneous	Preeclampsia	Survive
Sample#9	1,000	28	No	10	2A	Cesarean section	Eclampsia	Survive

NEC, necrotizing enterocolitis; BW, birth weight; GA, gestational age; SGA, small for gestational age; MOD, mode of delivery.

### The relationship between biomarker values in the first and second samples

In the first sample, the median (range) values of GFAP [1.40 (0.20–6.50) vs. 0.30 (0.10–1.30) *P* = 0.014], GDNF [2.84 (1.05–14.11) vs. 1.56 (1.07–3.48) *P* = 0.050], and FABP-2 [621.70 (278.40–2,207.00) vs. 294.20 (211.40–597.50) *P* = 0.002] in the group with NEC were significantly higher than those in the group without NEC. In the second sample, the median (range) values of GFAP [2.40 (0.30–3.10) vs. 0.30 (0.10–0.60) *P* = 0.003], GDNF [2.99 (0.56–10.30) vs. 1.46 (0.85–2.24) *P* = 0.019], and FABP-2 [646.8 (179.20–1,571.00) vs. 314.90 (184.70–521.60) *P* = 0.040] in the group with NEC were significantly higher than those in the group without NEC.

In infants with NEC, the median (range) values of GFAP [2.40 (0.30–3.10) vs. 1.40 (0.20–6.50) *P* = 0.767], GDNF [2.99 (0.56–10.30) vs. 2.84 (1.05–14.11) *P* = 0.859], and FABP-2 [646.80 (179.20–1,571.00) vs. 621.70 (278.40–2,207.00) *P* = 0.953] in the second sample were higher than those in the first sample. However, the increase was not statistically significant.

In infants without NEC, the median (range) values of GFAP [0.30 (0.10–0.60) vs. 0.30 (0.10–1.30) *P* = 0.749] in the second sample were similar than those in the first sample, the median (range) values of GDNF [1.46 (0.85–2.24) vs. 1.56 (1.07–3.48) *P* = 0.114] in the second sample were lower than those in the first sample, and the median (range) values of FABP-2 [314.90 (184.70–521.60) vs. 294.20 (211.40–597.50) *P* = 0.741] in the second sample were higher than those in the first sample. However, the results of the *P*-value were not statistically significant. The data is depicted in Table 3.

**Table 3:** Description of biomarkers in the group of infants with NEC and without NEC.

Biomarker	With NEC (*n* = 9)					Without NEC (*n* = 21)				
	Min	Max	Med	Mean	SD	Min	Max	Med	Mean	SD
**GFAP (ng/ml)**										
First sample	0.20	6.50	1.40	2.00	2.25	0.10	1.30	0.30	0.38	0.27
Second sample	0.30	3.10	2.40	1.74	1.29	0.10	0.60	0.30	0.34	1.16
**GDNF (ng/ml)**										
First sample	1.05	14.11	2.84	4.39	4.19	1.07	3.48	1.56	1.69	0.56
Second sample	0.56	10.30	2.99	3.94	3.26	0.85	2.24	1.46	1.30	0.37
**FABP-2 (ng/L)**										
First sample	278.40	2,207.00	621.70	844.44	677.04	211.40	597.50	294.20	335.48	104.39
Second sample	179.20	1,571.00	646.80	807.37	534.03	184.70	521.60	314.90	323.41	85.41

NEC, necrotizing enterocolitis; GFAP, glial fibrillary acidic protein; GDNF, glial-derived neurotrophic factor; FABP-2, fatty acid-binding protein-2; Min, minimum; Max, maximum; Med, median; SD, standard deviation.

**Figure 2:**
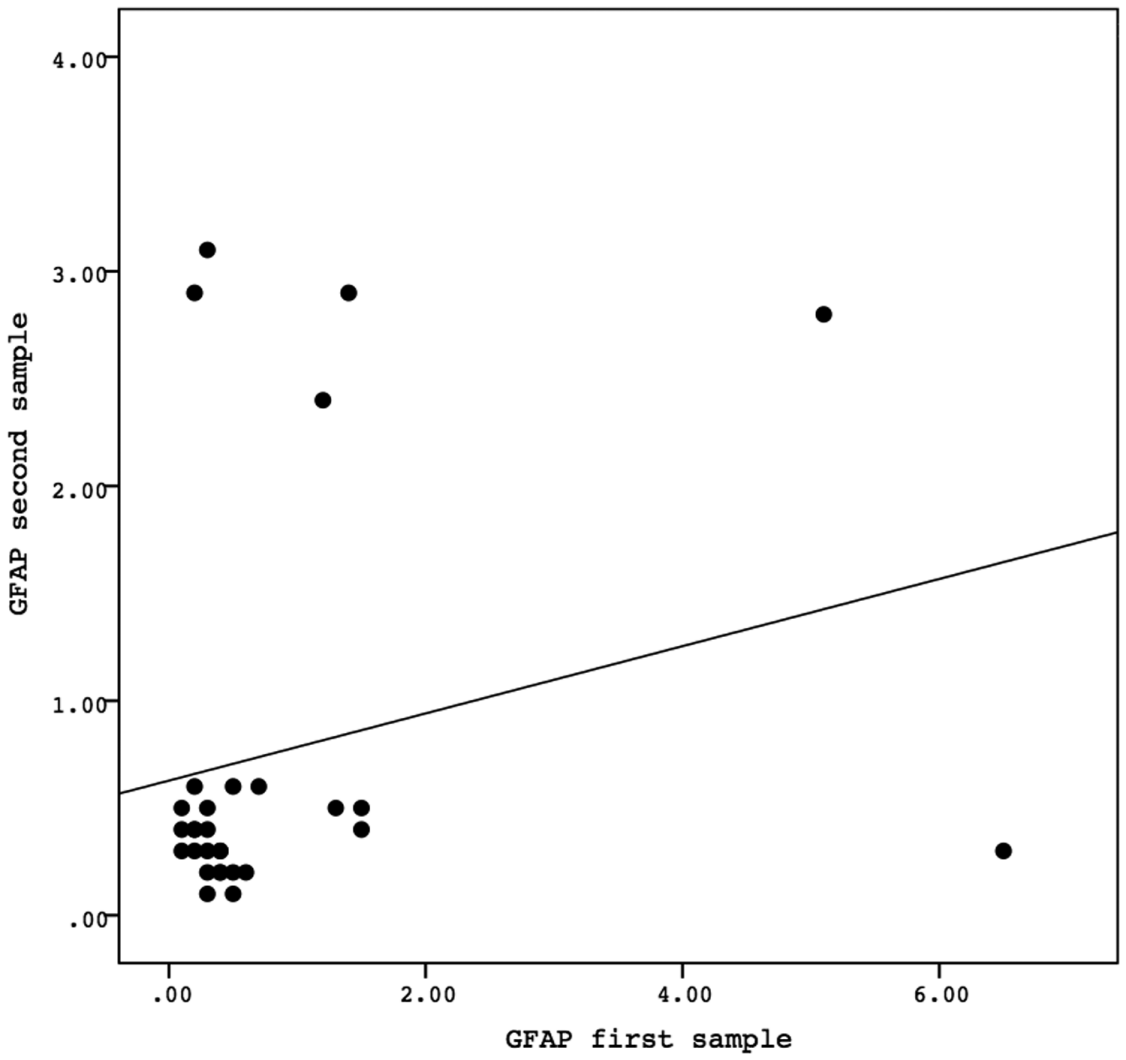
The correlation between the GFAP values of the first sample and the second sample. Correlation analysis using Spearman correlation demonstrated the correlation coefficient (*r*) = 0.081 and *P*-value = 0.669. GFAP, glial fibrillary acidic protein.

**Figure 3:**
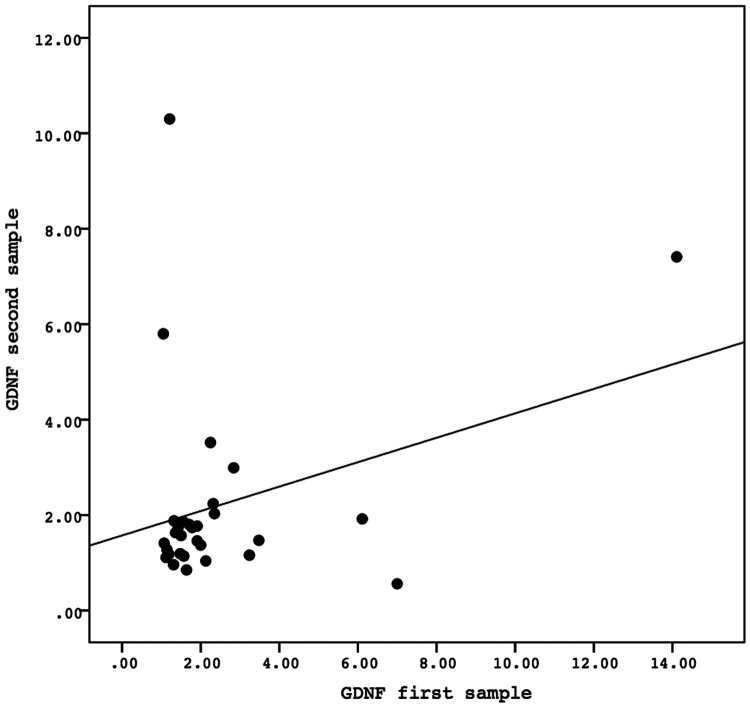
The correlation between the GDNF values of the first sample and the second sample. Correlation analysis using Spearman correlation demonstrated the correlation coefficient (*r*) = 0.138 and *P*-value = 0.468. GDNF, glial-derived neurotrophic factor.

**Figure 4:**
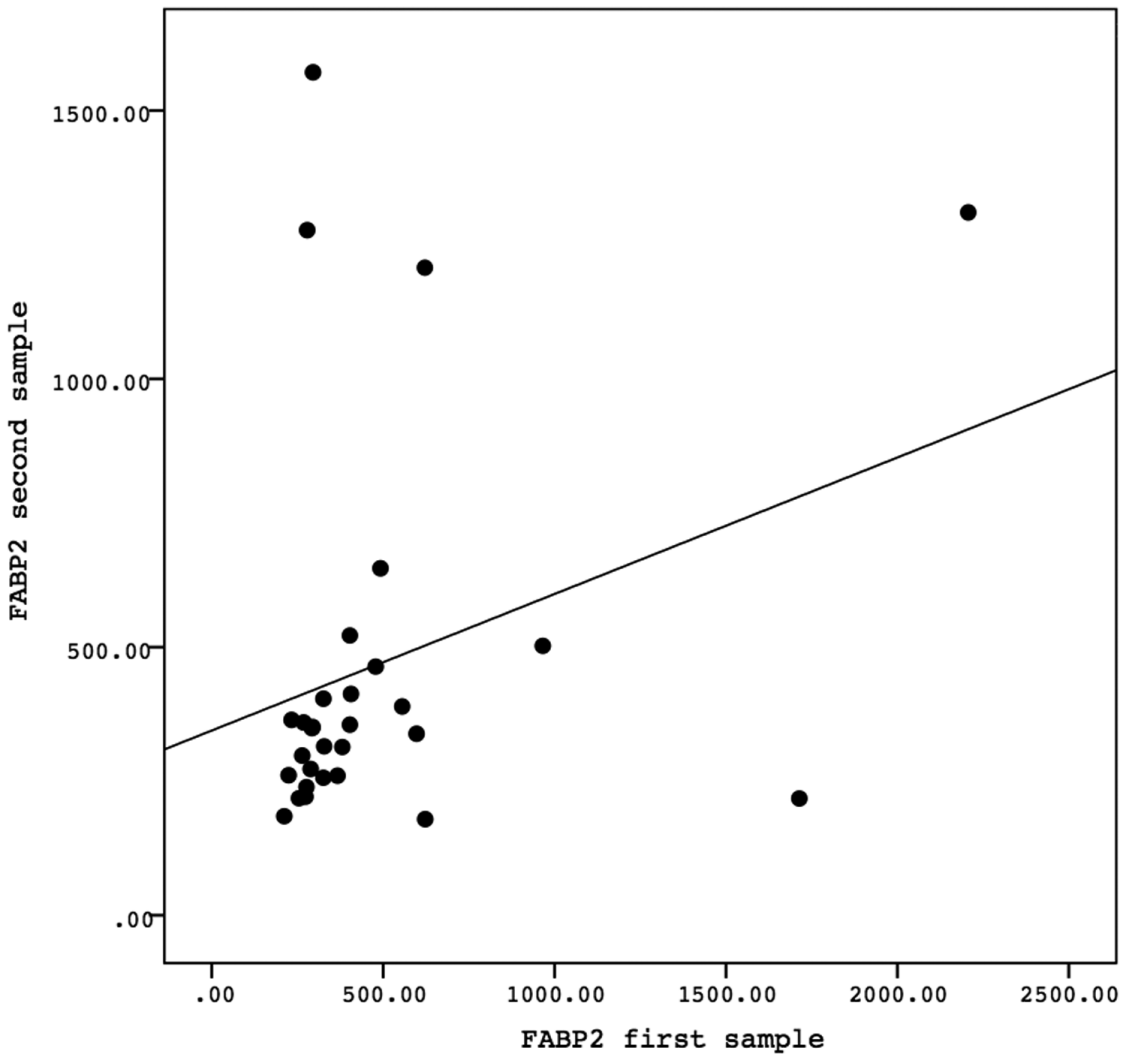
The correlation between the FABP-2 values of the first sample and the second sample. Correlation analysis using Spearman correlation demonstrated the correlation coefficient (*r*) = 0.346 and *P*-value = 0.061. FABP-2, fatty acid-binding protein-2.

A Spearman correlation analysis was carried out to investigate the relationship between the first and second samples. There was a positive correlation in the values of the biomarkers GFAP, GDNF, and FABP-2 between the first and second samples, although the results were not statistically significant. The correlation between the GFAP values of the first sample and the second sample demonstrated the correlation coefficient (*r*) = 0.081 and *P* = 0.669. The correlation between the GDNF values of the first sample and the second sample showed the correlation coefficient (*r*) = 0.138 and *P* = 0.468. Meanwhile, the correlation between the FABP-2 values of the first sample and the second sample revelead the correlation coefficient (*r*) = 0.346 and *P* = 0.061. The data is presented in Figures 2–4.

### The relationship between biomarker values at birth and incidence of NEC

This study performed a logistic regression analysis on each of the biomarker values at birth to investigate the risk of developing NEC. Statistical results showed that GFAP and FABP-2 at birth significantly associated with increased risk of NEC with OR = 15.629 (95% CI = 1.697–143.906), *P* = 0.015 and OR = 1.008 (95%CI = 1.001–1.015), *P* = 0.033, respectively. The results are described in Table 4.

**Table 4:** Logistic-regression analysis of biomarker values at birth on the risk of NEC.

Biomarker	OR	95% CI	*P*-value^a^
GFAP	15.629	1.697–143.906	0.015*
GDNF	2.654	0.919–7.666	0.071
FABP-2	1.008	1.001–1.015	0.033*

OR, Odds Ratio; CI, Confidence Interval; NEC, necrotizing enterocolitis; GFAP, glial fibrillary acidic protein; GDNF, glial-derived neurotrophic factor; FABP-2, fatty acid-binding protein-2.

^a^ Statistical analysis using logistic-regression.

* Significant if *P*-value < 0.05.

## Discussion

All research subjects in this study were preterm infants with gestational age <34 weeks and birth weight <1,500 g. These subject criteria were selected based on previous literature, which stated that most cases of NEC occurred in very-low-birth-weight infants (21). However, this study showed that infants with birth weight <1,000 g were more at risk for developing NEC. In addition, all NEC infants were singleton pregnancies, and most were male. Socio-economic factors also contribute to increasing the risk of NEC, including male gender, Black race, unclear marital status, maternal age <19 years, singleton pregnancy, and pregnancies of more than four times (22, 23). This study reported that approximately 30% of very low birth weight infants experienced NEC, whereas the mortality rate for NEC was 22%. These results are consistent with those found in other studies (3, 24), thus supporting the fact that NEC is still a threat to be aware of.

This study limits the length of observation for the presence of NEC to 14 days. The reason for this time duration was that we intended to analyze the association of early-onset NECs with perinatal conditions because we suspected that perinatal conditions were directly related to early-onset NECs. Previous studies have also found evidence of the role of prenatal and perinatal hypoxia-ischemia in the pathogenesis of early-onset NEC (16, 25). The classification of early-onset NEC and late-onset NEC was discussed by Yee et al. (26), who showed that the population of early-onset NEC was more common in infants with higher birth weight, higher gestational age, and vaginal delivery. Higher gestational age reflected more mature babies who tended to be given more aggressive enteral nutrition, so there was a greater potential for NEC.

The results of this study indicate that most infants with NEC have a history of cesarean delivery. These results are different from other studies which show that cesarean delivery is a protective factor against the occurrence of NEC (27, 28). This difference is probably due to the higher number of cesarean deliveries in Indonesia compared to other countries (29, 30).

All infants with NEC in this study had a history of Apgar score <7 at 5 min after birth. Apgar score <7 at 5 min can be considered as a possible sign of hypoxic-ischemic event at perinatal period. Previous studies have shown that the association between hypoxic-ischemic event and NEC has varied results. Hypoxic-ischemic event and diving reflex are NEC concepts that are traditionally understood (31). Primary hypoxic-ischemic event alone is considered not enough to be a risk factor for the presence of NEC. A multifactorial cause is needed for NEC, including abnormalities of the microvascular system, microbial translocation, inflammatory processes, immaturity of the immune response, genetic predisposition, and enteral nutrition (32).

This study conducted a logistic regression test to analyze how the biomarkers GFAP, GDNF, and FABP2 provide a risk for the occurrence of NEC. Logistic regression analysis showed significant results on the biomarkers GFAP and FABP-2. A meta-analysis study explained that FABP-2 has high accuracy in detecting NEC through blood and urine specimens (33). This is because the size of the FABP-2 molecule is very small, so if there is damage to the enterocytes, it will be easily released into the circulation, into the glomerulus, and secreted through the urine. FABP-2 detected in urine indicates the development of progressive NEC disease (33). In the case of intestinal injury lasting less than two hours, the villi are affected first while the crypts are intact. FABP-2 is expressed in villi. Thus, FABP-2 can be used for early detection (34). High levels of FABP-2 were found in infants following cardiopulmonary bypass indicating early enterocyte injury that potentially increases the risk of NEC (35). The FABP-2 assay in this study used umbilical blood immediately after birth, reflecting perinatal conditions. Previous studies reported that the umbilical blood FABP-2 of intrauterine growth retardation (IUGR) infants with abnormal antenatal Doppler had higher values than IUGR infants with normal antenatal Doppler (360.11 pg/ml vs. 285.12 pg/ml). The antenatal Doppler examination was performed seven days before delivery (34). This means that disturbances in fetal circulation during pregnancy will impact the baby's digestive tract condition during the perinatal period (16). This explains the high FABP-2 levels from birth in this study and its association with NEC occurrence within two weeks of birth. This study also found that the trend of FABP-2 levels after birth was slightly increased, although the increase was not statistically significant. These findings suggest that the inflammatory process mainly occurs in the perinatal period, although clinical manifestations are seen within two weeks. Changes in FABP-2 levels appear to precede clinical manifestations, indicating that FABP-2 levels at birth can be used as a predictor of early-onset NEC.

This study also found that high GFAP values at birth were associated with the NEC occurrence in early birth. GFAP is a specific marker for glial cells and is released when cell injury or death occurs (36). Enteric glial cells (EGC) can be found in the myenteric and submucosal plexuses involved in the ENS (37). Histopathological findings in NEC are edema, ischemia, and necrosis along the terminal ileum and proximal colon, extending to the submucosa, resulting in damage to plexus architecture. Any damage to the plexus architecture will cause mucosal and submucosal innervation loss. This neural abnormality in the NEC initiates the inflammatory process by activating the EGC (7). In another study, GFAP assays were used to detect glial cell damage in the brain and indicate hypoxic-ischemic encephalopathy (9). The presence of an increase in GFAP in the NEC indicates a breakdown of the blood-brain barrier and selective neuronal degeneration. This shows that NEC is correlated with central nervous system damage that has the potential to cause neurodevelopmental disorders (38). In this study, head ultrasound or magnetic resonance imaging (MRI) was not performed on the infants, so whether the increase in GFAP was due to brain damage could not be explained. However, infants with NEC in this study had a history of Apgar score <7 at 5 min, so this finding supports the concept of a gut-brain axis that even occurs during the perinatal period (39).

GDNF is a mediator molecule secreted by epithelial enterocytes and EGC. GDNF plays a role in motility, endocrine secretion, and the immune system in the gut. GDNF is also responsible for interacting with enteric neuron cells and the intestinal epithelium. The role of GDNF is to improve the function of intestinal tight junctions through stimulation of claudin-1, claudin-5, and occludin; therefore, the role of GDNF should be to repair intestinal damage and stimulate intestinal maturation (40). However, this study obtained the opposite result, which found that increased GDNF increased the risk of developing NEC. In vitro studies show that ischemia will damage intestinal tight junctions, increase occludin expression, and increase FABP-2 expression. Intestinal damage at the tight junction level changes the enterocyte defense function (41, 42). Another study also showed that tight junction damage was found in NEC stimulated by hypoxia/re-oxygenation as indicated by increased expression of tight junction proteins, including claudin-1 and occludin as well as increased expression of FABP-2. That study showed that tight junctions are involved in the pathogenesis of NEC (43). In this study, we speculated that GDNF could stimulate FABP-2 through tight junction proteins such as occludin, claudin-1, or claudin-5, which requires further research.

This study carried out logistic regression analysis to analyze GFAP, GDNF, and FABP-2 at birth in the presence of NEC. When compared between GFAP and FABP-2, an increase in GFAP has a higher risk of developing NEC than an increase in FABP-2 (15 vs. 1). FABP-2 is a protein located in the cytoplasm of enterocytes and is released when disruption of enterocytes occurs (44). A very high increase in FABP-2 may indicate a large area of the intestine affected (45). In severe NEC, the damage can be deeper up to the submucosal level. In this condition, there will be plexus damage and an increase in GFAP (7). In addition, in severe NEC, tight junction proteins may be involved (42).

This study has several limitations that need to be considered. This study did not perform a head ultrasound or MRI, because we did not perform routine head ultrasound in every preterm infants due technical and logistic limitation. So we could not exclude the possibility of an increase in GFAP caused by brain injury. In addition, this study did not include extended antenatal data, so it could not explain that some other maternal comorbid factors associated with intestinal injury in the perinatal or prenatal period. Therefore, studies of maternal comorbid involvement with perinatal intestinal injury are warranted. Based on our nutrition protocol, enteral nutrition was given using human milk or preterm formula if human milk was not available. However, previous studies have shown that exclusive breastfeeding is a protective factor against the occurrence of NEC (3, 22). Most of the very low birth weight infants born in our hospital received enteral nutrition which was a combination of breast milk and premature formula because their mother was still critically ill. In addition, our hospital has not yet had standard regulations regarding donor breast milk. Therefore, the role of premature formula in the occurrence of NEC in this study could not be ruled out. Another limitation was the low number of the study participants. More study with higher number of participants is warranted. Moreover, further studies on the impact of NEC in early life on future development are needed.

## Conclusion

This study found that increased GFAP, GDNF, and FABP-2 at birth were associated with NEC occurrence within two weeks of birth. These findings suggest that early-onset of NEC is associated with an intestinal injury that has occurred since the perinatal or even prenatal period. Early detection is required in NEC so that severe morbidity can be prevented. A comprehensive strategy is now needed to prevent neonatal NEC involving practices beginning in the perinatal and prenatal period.

## Data Availability

The original contributions presented in the study are included in the article/Supplementary Material, further inquiries can be directed to the corresponding author/s.
